# Molecular Evolutionary Dynamics of Respiratory Syncytial Virus Group A in Recurrent Epidemics in Coastal Kenya

**DOI:** 10.1128/JVI.03105-15

**Published:** 2016-04-29

**Authors:** James R. Otieno, Charles N. Agoti, Caroline W. Gitahi, Ann Bett, Mwanajuma Ngama, Graham F. Medley, Patricia A. Cane, D. James Nokes

**Affiliations:** a Epidemiology and Demography Department, Kenya Medical Research Institute–Wellcome Trust Research Programme, Kilifi, Kenya; b Department of Biomedical Sciences, Pwani University, Kilifi, Kenya; c Department of Global Health and Development, London School of Hygiene and Tropical Medicine, London, United Kingdom; d Public Health England, Salisbury, United Kingdom; e School of Life Sciences and WIDER, University of Warwick, Coventry, United Kingdom

## Abstract

The characteristic recurrent epidemics of human respiratory syncytial virus (RSV) within communities may result from the genetic variability of the virus and associated evolutionary adaptation, reducing the efficiency of preexisting immune responses. We analyzed the molecular evolutionary changes in the attachment (G) glycoprotein of RSV-A viruses collected over 13 epidemic seasons (2000 to 2012) in Kilifi (*n* = 649), Kenya, and contemporaneous sequences (*n* = 1,131) collected elsewhere within Kenya and 28 other countries. Genetic diversity in the G gene in Kilifi was dynamic both within and between epidemics, characterized by frequent new variant introductions and limited variant persistence between consecutive epidemics. Four RSV-A genotypes were detected in Kilifi: ON1 (11.9%), GA2 (75.5%), GA5 (12.3%), and GA3 (0.3%), with predominant genotype replacement of GA5 by GA2 and then GA2 by ON1. Within these genotypes, there was considerable variation in potential *N*-glycosylation sites, with GA2 and ON1 viruses showing up to 15 different patterns involving eight possible sites. Further, we identified 15 positively selected and 34 genotype-distinguishing codon sites, with six of these sites exhibiting both characteristics. The mean substitution rate of the G ectodomain for the Kilifi data set was estimated at 3.58 × 10^−3^ (95% highest posterior density interval = 3.04 to 4.16) nucleotide substitutions/site/year. Kilifi viruses were interspersed in the global phylogenetic tree, clustering mostly with Kenyan and European sequences. Our findings highlight ongoing genetic evolution and high diversity of circulating RSV-A strains, locally and globally, with potential antigenic differences. Taken together, these provide a possible explanation on the nature of recurrent local RSV epidemics.

**IMPORTANCE** The mechanisms underlying recurrent epidemics of RSV are poorly understood. We observe high genetic diversity in circulating strains within and between epidemics in both local and global settings. On longer time scales (∼7 years) there is sequential replacement of genotypes, whereas on shorter time scales (one epidemic to the next or within epidemics) there is a high turnover of variants within genotypes. Further, this genetic diversity is predicted to be associated with variation in antigenic profiles. These observations provide an explanation for recurrent RSV epidemics and have potential implications on the long-term effectiveness of vaccines.

## INTRODUCTION

Human respiratory syncytial virus (RSV) is a leading worldwide viral cause of lower respiratory tract infection (LRTI) in both young children and the elderly and is a frequent cause of hospitalization (1–3). Despite considerable effort to develop an RSV vaccine, none has been licensed. The virus is characterized into two groups (A and B) (4, 5). Strains within the groups can be further classified into genotypes and variants based on genetic divergence (6–9). The RSV attachment (G) glycoprotein is responsible for virus binding to the host cell surface receptor and is a target of human neutralizing antibodies, together with the fusion (F) glycoprotein (10, 11). The G protein shows the highest genetic and antigenic variability among all the RSV structural proteins, with evidence of accumulation of amino acid changes in its hypervariable regions over time (6, 12, 13), making its study relevant in vaccine development strategies.

The dynamics of RSV infections have been followed globally, in communities, in families, and in individuals over time (7, 14, 15). RSV epidemics often occur at regular intervals in communities, usually annually (16, 17). In Kilifi, RSV epidemics usually start between September/November of one year, peaking between January and March, with infections continuing to be detected until June/August of the following year. Individuals are found to be repeatedly reinfected with RSV throughout their lifetimes (18). The driving factors that facilitate these patterns are poorly understood. The two RSV groups have been shown to cocirculate in epidemics, with RSV-A generally occurring more frequently than RSV-B (19, 20). Additionally, sequence data have shown that a local RSV epidemic may be characterized by multiple genotypes and variants and that new variants may replace older ones in subsequent epidemics (7–9). These patterns indicate that the circulation of strains varying from those that have recently caused community epidemics is favored. A better understanding of the interplay between the virus epidemiology, the genetic and antigenic variability, and host immune dynamics in producing new RSV community epidemics may contribute to better design of control measures.

It has been suggested that genetic variability and antigenic variation within the G protein contributes to the propensity of the virus to cause repeated individual reinfections and recurrent epidemics in communities by aiding evasion of host preexisting immune responses (21). Particular amino acid substitutions and changes in glycosylation profile of the G protein have been shown to profoundly affect the antigenic profile of the virus (22, 23). The present report analyzes the genetic variability and molecular evolution of RSV group A viruses identified over 13 RSV epidemic seasons (2000 to 2012) at the rural Kenyan Coast and contemporaneous sequences collected elsewhere within Kenya and 28 other countries. Close to half of the total group A detections during the period were sequenced in their G ectodomain region. We explored the patterns of RSV-A genetic diversity in Kilifi, the influence of the observed genetic changes on the resultant protein molecule and RSV epidemiology, and the placement of the locally detected viruses on the global phylogeny.

## MATERIALS AND METHODS

### Study population and samples.

The study was undertaken in Kilifi County, which is located at the Kenyan Coastal region with a predominantly rural catchment population of around one million. This was part of a surveillance study that aimed at understanding the epidemiology and disease burden of RSV-associated pneumonia cases (24). In this study, we used three sources of clinical samples: (i) LRTI cases for children <5 years admitted to Kilifi County Hospital (KCH) collected over the period from 2000 to 2012 (3, 24), (ii) KCH outpatient presentations for children <5 years with acute respiratory infection (ARI) from April to August 2002 (25, 26), and (iii) the Kilifi RSV birth cohort (KBC) ARI cases identified between January 2002 to August 2003 (25, 27). Nasopharyngeal aspirates, nasal washes, or nasopharyngeal swabs were collected for inpatient samples, while nasal washes were collected for the outpatient and community sampling of the birth cohort. All sample sets arise from the same catchment population and were processed similarly in the laboratory and in downstream analyses.

### Sample processing and sequencing.

RSV detection was conducted by antigen (immunofluorescence antibody test [IFAT]) and nucleic acid (reverse transcriptase real-time PCR) tests as previously described (24, 27, 28). For the hospital inpatient RSV-A samples collected during the surveillance period from 2002 to 2012, we randomly sampled ≥50% of the archived RSV IFAT-positive samples from each epidemic for processing. The additional hospital inpatient (2000 to 2001), outpatient (2002), and Kilifi RSV birth cohort (2002 to 2003) samples included in this analysis had previously been sequenced and preliminary results reported elsewhere (25, 27).

Total viral RNA was extracted using QIAamp viral RNA minikit (Qiagen) according to the manufacturer's instructions. Reverse transcription was performed using random primers and the OmniScript RT kit (Qiagen). The cDNA was amplified with previously described primers targeting the G ectodomain region (21, 25). BigDye v3.1 chemistry was used on an ABI 3130xl instrument to sequence the amplicons. The sequence reads were assembled into contigs using Sequencher v5.0.1 (Gene Codes Corporation, USA) and Gap4 release 2.0.0b9 (29).

### Global comparison data set.

All RSV-A G-gene sequences deposited in GenBank as of 20 November 2015 of the same length or longer to the Kilifi sequences and collected between 2000 and 2012 were collated and phylogenetically compared with the Kilifi sequences. This analysis aimed to determine the relatedness of the Kilifi viruses to those circulating around the world and thereby understand their global context. A total of 1,415 sequences from 29 countries were used in this analysis; 1,075 sequences from 28 countries and 340 sequences from Kenya. These sequences had been filtered from a larger data set of 1,792 sequences to include only the unique sequences per country except for Kenya where sequences from Kilifi (*n* = 284) and the rest of the country were filtered separately. In addition, the unique sequences from Kilifi were subsampled per epidemic since we had precise dates of collection (day/month/year), as opposed to the global data set, where we subsampled each country collectively from 2000 to 2012 since most of the global sequences either only had the year of collection or the samples were too sparse to sample by year. Unique sequences were identified as sequences that differ by at least one nucleotide from any other sequence over the sequenced region.

### Sequence alignments and diversity analysis.

All the sequences were collated and aligned using MAFFT alignment software v7.220 (30) with manual editing in Se-Al software v2.0a11 (31). Nucleotide and amino acid variability within genotypes and for all Kilifi sequences were calculated for individual epidemics and for the entire period (2000 to 2012). The analyses used MEGA v6.06 (32) for amino acid variability (p-distance) and in DnaSP v5.10 (33) for nucleotide variability (Pi [π]).

### Phylogenetic analyses.

The best-fit nucleotide substitution model was determined using jModelTest v2.1.7 (34). Maximum-likelihood (ML) phylogenetic trees were inferred by MEGA 6.06 under the general-time-reversible (GTR) model with site heterogeneity gamma (G) model, four gamma categories, and a proportion of the sites invariable (I) (32). Bootstrapping with 1,000 iterations was implemented to evaluate branch support of the phylogenetic clusters generated.

RSV-A genotypes were designated as previously described by Peret et al. (8). A cluster was defined as a virus or group of viruses within the same genotype that fall(s) into a phylogenetic subset away from the rest with >70% bootstrap statistical support.

### Variant introduction and persistence analysis.

Using a definition recently developed to characterize variants within epidemics for RSV group B viruses (9), we determined the number of variants circulating in Kilifi between 2000 and 2012 for the group A viruses. We used the same definition considering that previous estimates of the rates of nucleotide substitution for the G-gene in RSV-A and RSV-B are similar, i.e., 1.83 × 10^−3^ and 1.95 × 10^−3^ nucleotide substitutions/site/year, respectively (35, 36). The variant designation was given to a group of viruses (where the group includes a singleton) within a genotype that possesses ≥4 nucleotide differences in the G ectodomain region compared to other viruses. This analysis was done using usearch v8.1.1756 (37).

### Evolutionary analysis.

Evolutionary analysis used the Bayesian Markov Chain Monte Carlo-based approach implemented in BEAST v1.8.2 (38), assuming an uncorrelated lognormal relaxed molecular clock model prior to accommodate variation in molecular evolutionary rate among lineages. Input sequences were tip-dated with day, month and year of collection. We implemented the GTR (G) nucleotide substitution model of evolution and gamma (Γ) plus invariant (I) site heterogeneity model. The demographic history of the viral populations was modeled using the nonparametric Gaussian Markov random-fields (GMRF) Bayesian Skyride model (39). The analysis was run through 50 million steps with sampling after every 2,500 steps. BEAST run convergence confirmation (effective sample size [ESS] > 200) and analysis of parameter estimates were done using Tracer v1.6 (http://tree.bio.ed.ac.uk/software/tracer/). The BEAST trees were summarized using TreeAnnotator v1.8.2 (http://beast.bio.ed.ac.uk/treeannotator), and the maximum clade credibility tree was visualized by FigTree v1.4.0 (http://tree.bio.ed.ac.uk/software/figtree/).

### Diversifying selection analyses.

RSV-A genotype nucleotide alignments were analyzed for positive selection using PAML v4.8 (40). Data for each genotype were analyzed using the site models M0 (one-ratio), M1a (neutral), M2a (selection), and M3 (discrete). The M0 model calculates one nonsynonymous/synonymous *dN/dS* ratio (ω) for all sites, i.e., averaged over all codon sites and over the entire evolutionary time that separates the sequences. The remaining three models (M1a, M2a, and M3) allow for heterogeneous ratios among sites. M1a assumes two categories of sites: conserved sites with ω_0_ = 0 and neutral sites with ω_1_ = 1. M2a adds a third category of sites, positively selected sites (ω_2_ > 1), which is estimated from the data. M3 models the three heterogeneous ratios among sites as M2a but using an unconstrained discrete distribution (41).

The models M0 and M1a were used to test for selection over the whole sequenced region, while the remaining models tested for the presence of positively selected sites (ω > 1). Both the Naive Empirical Bayes and Bayes Empirical Bayes methods were used to identify the sites under adaptive evolution (42).

### Protein substitution analysis.

The gain and loss of *N*-glycosylation and *O*-glycosylation sites were predicted using the NetNGlyc 1.0 and NetOGlyc 4.0 servers (43, 44). Only the default Asn-X-Ser/Thr sequon (where “X” is not proline) was considered in *N*-glycosylation prediction. Patterns of change in amino acids at all positions of the sequences were analyzed using python scripts.

### Temporal epidemic patterns analysis.

Epidemic seasons were designated to begin 1 September of one year and end 31 August of the following year. The dominant RSV group within a given epidemic was associated with ≥65% of the cases; otherwise, the groups were codominant.

### Ethics statement.

The samples obtained in Kilifi were collected following informed written consent from each child's guardian or parent. KEMRI Ethical Review Board, Kenya, and the Coventry Research Ethics Committee of the UK approved the study protocols (3, 24).

### GenBank accession numbers.

The newly generated sequences analyzed here have been deposited in GenBank under accession numbers KT765213 to KT765836. The previously sequenced and reported RSV-A sequences from Kilifi added to this analysis had been archived in GenBank under accession numbers AY524573 to AY524663 and AY660667 to AY660684.

## RESULTS

A total of 2,135 RSV positive clinical samples from hospital surveillance over 13 epidemic seasons (2000 to 2012) were examined. Overall, 1,246 (58.4%) of the samples were RSV-A, 684 (32%) RSV-B, 28 (1.3%) mixed infections (RSV-A/B), and 177 (8.3%) untyped/unclassified. RSV-A viruses were detected in all the 13 epidemics with the proportion of samples being designated group A in each epidemic ranging from 12.9 to 93.7% (see Table S1 in the supplemental material).

A total of 649 RSV-A G gene nucleotide sequences were available for further analysis: 564 from hospital inpatient surveillance from 2002 to 2012, 60 sequences from an earlier collection of in- and outpatient samples collected in Kilifi from 2000 to 2002, and 25 sequences from the Kilifi RSV birth cohort (27). The sequences were 618 to 690 nucleotides in length corresponding to nucleotides 295 to 912 of the reference strain A2 (M74568). Of the 649 sequences, 284 (43.8%) were unique. The number of duplicates per sequence ranged between 2 and 37 sequences. From the 284 unique sequences that had been subsampled per epidemic, we further collectively analyzed this sequence set for uniqueness. We found only 20 sequences of the 284 that were identical across epidemics, i.e., 264 sequences were unique across all the 13 epidemics differing by at least one nucleotide.

### Diversity within and between epidemics and genotypes.

**FIG 1:**
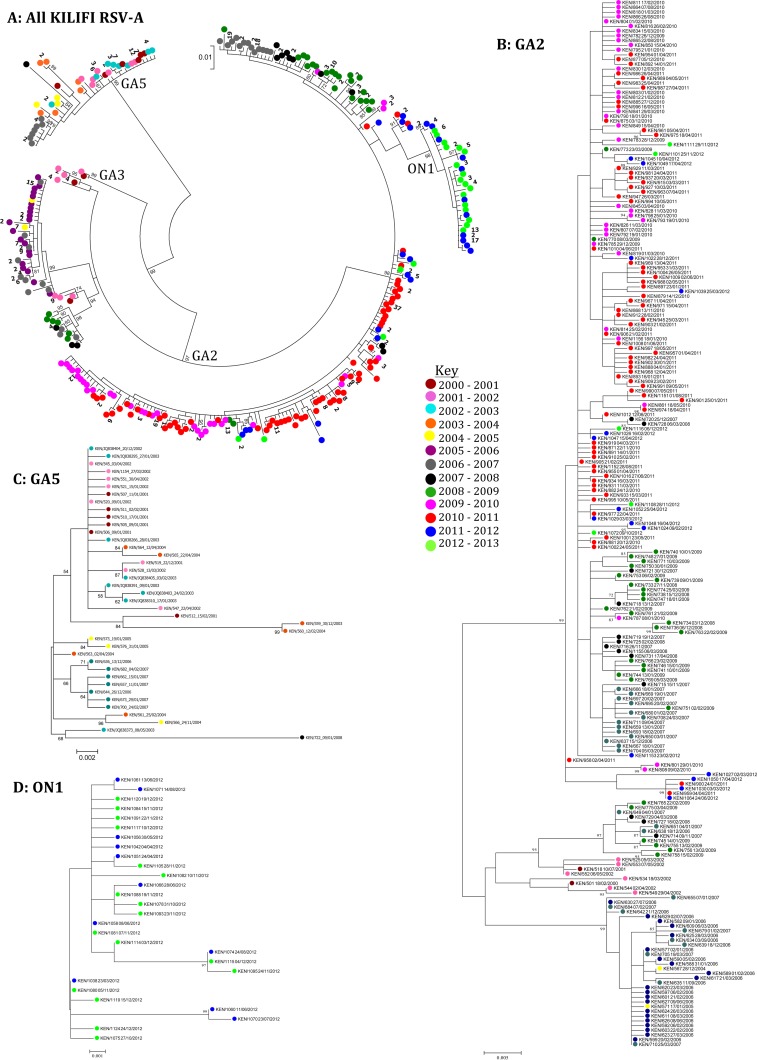
ML phylogenetic tree of 284 unique RSV group A G-gene, ectodomain sequences obtained in Kilifi, Kenya, from 2000 to 2012. The taxa are color coded by epidemic season of detection, as shown by the key. Panel A represents all the 284 sequences, with the numbers in boldface next to the colored circles indicating the number of identical sequences to the one shown. Genotypes, as assigned by Peret et al. (8), are shown for the main branches and only bootstrap values >70% are shown. Panels B, C, and D are subtrees from panel A representing genotypes GA2, GA5, and ON1, respectively. The taxon names denote country/serial number_date. The scale bars indicate nucleotide substitutions.

**FIG 2:**
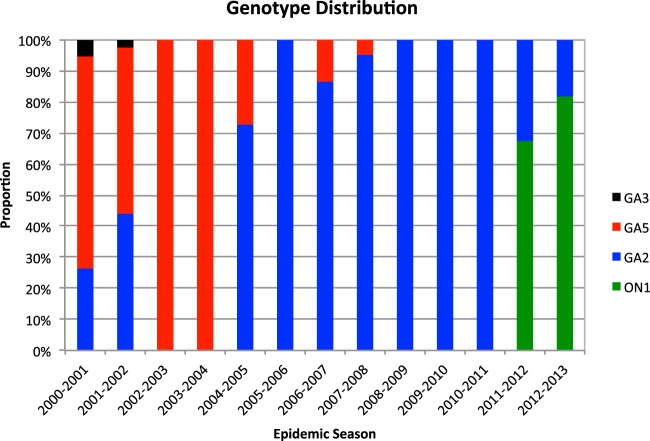
RSV-A genotype distribution over 13 successive epidemic seasons in Kilifi, 2000-2001 to 2012-2013. The genotypes are shown in different colors: GA2 (blue), GA5 (red), GA3 (black), and ON1 (green).

The genetic relatedness of the 284 unique Kilifi RSV-A sequences is shown in an ML phylogenetic tree in Fig. 1A, with the taxa color coded according to the epidemic season. On this phylogeny, the analyzed sequences fell within three major clusters corresponding to genotypes GA5, GA3, and GA2. Within these genotypes, the sequences formed distinct clusters that frequently comprised sequences that had been sampled within the same epidemic. The recently emerged genotype ON1 that has a 72-nucleotide duplication within the G ectodomain region was most closely related to genotype GA2. The genotype prevalence pattern over the period is shown in Fig. 2, with overall detection as follows: GA2 (*n* = 490, 75.5%), GA3 (*n* = 2, 0.3%), GA5 (*n* = 80, 12.3%), and ON1 (*n* = 77, 11.9%). Genotype-specific phylogenies derived from the unique GA2, GA5, and ON1 sequences are shown in Fig. 1B to D.

Genotype GA2 was observed in 11 of 13 of the epidemics, including 4 where it was the only RSV-A genotype detected. The nucleotide and amino acid variability within the genotypes in each epidemic is summarized in Table 1. For GA2, the nucleotide sequences varied by a maximum of 2.04% within an epidemic and 2.27% over the entire period with corresponding amino acid variability of up to 3.75 and 3.66%, respectively. GA5 sequences were less frequently observed and were not seen after the 2007-2008 epidemic (Fig. 2). Sequences belonging to genotype ON1 were first seen globally in 2010 to 2011 with initial detection in Kilifi in the 2011-2012 epidemic, and 77 genotype ON1 sequences from Kilifi were found in 2012 (45, 46). Genotype GA3 was represented by just 2 unique sequences of the 58 sequences obtained between 2000 and 2002 and so will not be considered further in detail.

**TABLE 1:** Nucleotide and amino acid variability in RSV-A genotypes identified in Kilifi, Kenya, 2000-2001 to 2012-2013

Epidemic season	% Variability (no. of sequences)^*a*^					
	GA2 and ON1^*b*^		GA5		Overall	
	Nucleotides	Amino acids	Nucleotides	Amino acids	Nucleotides	Amino acids
2000-2001	0.78 (5)	1.17 (5)	0.30 (12)	0.81 (12)	4.26 (18)	8.56 (18)
2001-2002	1.52 (18)	2.66 (18)	0.25 (21)	0.22 (21)	4.99 (40)	9.49 (40)
2002-2003	– (0)	– (0)	0.45 (25)	0.67 (25)	0.45 (25)	0.67 (25)
2003-2004	– (0)	– (0)	1.76 (7)	3.11 (7)	1.76 (7)	3.11 (7)
2004-2005	0.12 (8)	0.24 (8)	0.97 (3)	1.63 (3)	4.72 (11)	8.30 (11)
2005-2006	0.38 (56)	0.83 (56)	– (0)	– (0)	0.38 (56)	0.83 (56)
2006-2007	1.90 (70)	2.93 (70)	0.32 (11)	0.36 (11)	3.75 (81)	6.26 (81)
2007-2008	1.71 (18)	3.31 (18)	NA (1)	NA (1)	2.62 (19)	4.97 (19)
2008-2009	2.04 (47)	3.75 (47)	–	–	2.04 (47)	3.75 (47)
2009-2010	0.50 (87)	0.70 (87)	–	–	0.50 (87)	0.70 (87)
2010-2011	0.66 (154)	0.99 (154)	–	–	0.66 (154)	0.99 (154)
2011-2012	1.39 (20)	2.76 (20)	–	–	1.59 (51)	3.37 (51)
	**0.43 (31)**	**1.02 (31)**		–		
2012-2013	1.16 (7)	0.98 (7)	–	–	0.99 (53)	1.59 (53)
	**0.37 (46)**	**0.46 (46)**				
Entire period	2.27 (567)	3.66 (567)	0.77 (80)	1.23 (80)	3.87 (649)	6.44 (649)

a Nucleotide diversity, Pi (π), was calculated using DnaSP v5.10 (33). Amino acid diversity, p-distance, was calculated using MEGA v6.06 (32). NA, not applicable. −, no sample.

b ON1 values are indicated in boldface.

**FIG 3:**
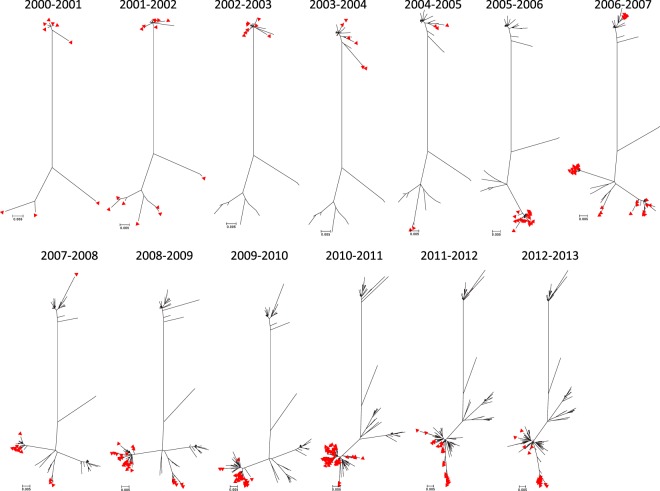
Accumulation of diversity in RSV-A strains over time as shown by ML radiation phylogenetic trees from Kilifi Kenya 2000-2001 to 2012-2013. Each panel shows RSV-A sequences observed since the start of the study with the sequences seen in each new epidemic highlighted with a red triangle. The clusters on the upper portion of the trees include genotype GA5 sequences, while those on the lower parts are GA2 and ON1 sequences. The lone branch in the middle includes genotype GA3 sequences.

Figure 3 illustrates the accumulation of diversity over time in the RSV-A sequences from Kilifi. It can be seen that although there was much accumulation of variation in the sequences, the sequences generally clustered closely with the sequences from previous years, with clear replacement of the previously predominant clusters with time.

### Dynamics of RSV-A variants in Kilifi.

**FIG 4:**
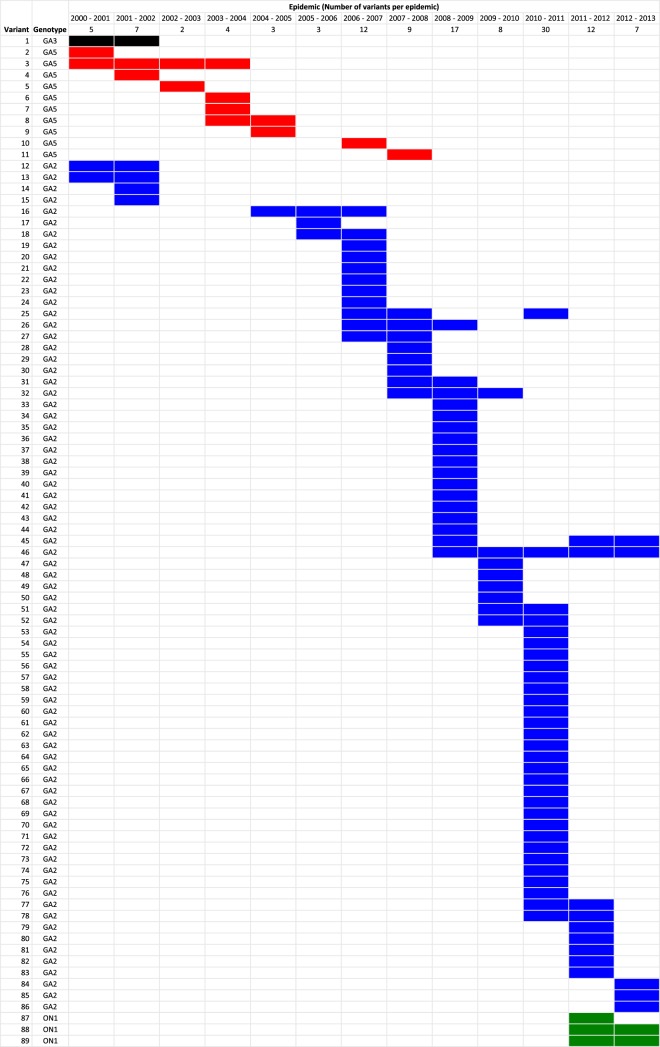
Temporal occurrence patterns of the 89 RSV-A variants (rows) detected within RSV epidemic seasons in Kilifi Kenya, 2000-2001 to 2012-2013. A variant was defined as a group of viruses (where group includes a singleton) within a genotype that possesses ≥4 nucleotide differences in the G ectodomain region compared to other viruses (see Materials and Methods). The genotypes are shown in different colors: GA2 (blue), GA5 (red), GA3 (black), and ON1 (green).

A total of 89 distinct variants were determined from 649 sequences (Fig. 4) on the basis of a single sequence or cluster of sequences with ≥4 nucleotide differences from other sequences in the data set. The variants comprised of between 1 and 119 sequences, 49.4% (44/89) of which were singletons. By genotypes, there were 75, 10, 3, and 1 variants for genotypes GA2, GA5, ON1, and GA3, respectively. All the epidemics were characterized by presence of multiple variants. However, only 22.5% (20/89) of the variants were observed in several (2 to 5) epidemics, with the remainder each observed in only one epidemic. Two of the variants observed in several epidemics were detected in nonconsecutive epidemics. As well, there were two variants that were observed over four consecutive epidemics.

### Rate of substitution and most recent common ancestor (MRCA).

Using Bayesian analysis, the rate of substitution for the Kilifi data set was estimated at 3.58 × 10^−3^ nucleotide substitutions/site/year (95% highest posterior density [HPD] interval = 3.04 × 10^−3^ to 4.16 × 10^−3^). By genotype, the rate of substitution was estimated at 2.61 × 10^−3^ (95% HPD = 2.22 × 10^−3^ to 3.02 × 10^−3^) for GA2, 2.89 × 10^−3^ (95% HPD = 1.29 to 4.49) for ON1, and 2.28 × 10^−3^ (95% HPD = 1.51 to 3.05) for GA5, with no significant difference in the rate between genotypes as the 95% HPD for each overlap. For the global data set, the substitution rate was estimated at 3.06 × 10^−3^ nucleotide substitutions/site/year (95% HPD = 2.78 to 3.35), while the MRCA was estimated to date back 43.9 years to 1968 (95% HPD = 31.3 to 58.3). The predicted demographic history of RSV-A in Kilifi (data not shown) was characterized by fluctuating effective population size, a measure of relative genetic diversity, a finding consistent with the continual RSV case detection across the years sampled.

### Amino acid variability and glycosylation.

**FIG 5:**
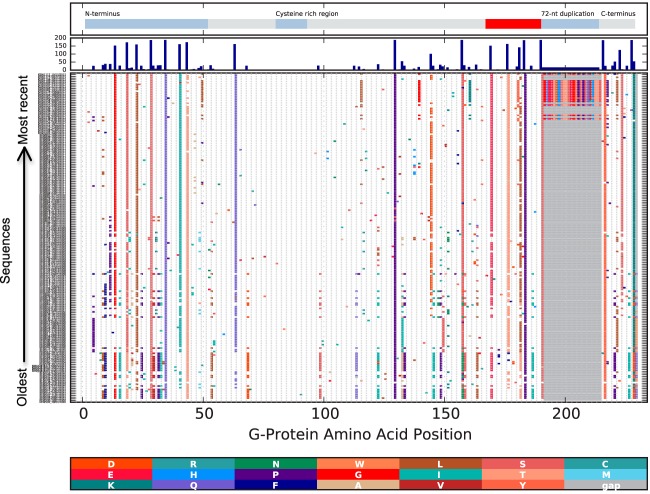
Changes in partial RSV-A G-protein for sequences isolated in Kilifi Kenya from 2000-2001 to 2012-2013. All unique protein sequences per epidemic were collated, aligned and the amino acid differences from the earliest sequence determined and marked with vertical colored bars, with the new amino acid residue color coded, as shown at the bottom. A gray bar indicates a gap in the query sequence. Sequences are ordered by sample collection date, with the earliest samples at the bottom of the graph. Indicated at the top of the graph are the functional domains of the G protein and the 72-nucleotide duplication of genotype ON1 (original sequence in “red” and duplicate in “light-blue steel”). The histogram in the second panel from the top indicates the total number of changes at each position.

The alignment of unique Kilifi RSV-A amino acid sequences, ordered by date of sampling, to show amino acid variability per site is given in Fig. 5. We identified 34 codon sites with amino acid substitutions that were distinct between the genotype GA2/ON1, GA5, and GA3 viruses identified in Kilifi. Although most (31/34) of these sites were shared between two genotypes and different in the other genotype, three of these sites had a unique amino acid for each genotype. Overall, 29 (36.3%) unique amino acid sequences were observed for genotype GA5 viruses and 183 (32.2%) for genotypes GA2 and ON1. The genotype GA5 amino acid sequences were all the same length, i.e., a predicted overall G protein of 299 amino acids. The genotype GA2 sequences were 298 or 299 amino acids in length due to usage of alternative stop codons. Sequences from ON1 genotype viruses had a 24-amino-acid insertion as previously described (45).

Twelve codon positions were predicted to be potentially *N*-glycosylated (103, 105, 135, 197, 237, 238, 242, 246, 250, 251, 273, and 294). Considerable variation was seen in the individual potential *N*-glycosylation sites and the resultant combined patterns. Genotypes GA2 and ON1 viruses together showed 15 different patterns (designated 2A to 2O) involving eight different possible sites. The minimum number of possible *N*-glycosylation sites per sequence was two, and the maximum was six. The site patterns and their occurrence in the epidemics are shown in Tables 2 and 3. It can be seen that new patterns are seen in every epidemic after 2004 until 2011, when the GA2 viruses start to decline in prevalence being replaced by the emerging ON1 genotype. Five patterns were seen in multiple epidemics of between two to five before disappearance, one pattern was observed in earlier epidemics before disappearance then recurrence, but most of the patterns (nine) were only seen in single epidemics. With respect to GA5, only three *N*-glycosylation patterns were seen (5A to 5C), with one pattern being predominant from 2000 to 2001 until 2004 to 2005, occurring again in 2006 to 2007, along with a second pattern, and the third pattern only being seen in 2007 and 2008. From 2008 to 2009 until from 2012 to 2013, genotype GA5 was not detected in Kilifi.

**TABLE 2:** Patterns of potential *N*-glycosylation sites for RSV-A sequence data from Kilifi, Kenya: genotypes GA2 and ON1

Designation code	Codon position^*a*^								Epidemic(s) observed
	103	135	197	237	246	251	273	294	
2A	+	+	–	+	–	+	–	+	2000-1, 2001-2, 2005-6, 2006-7, 2010-11, 2011-12
2B	+	+	–	+	–	+	+	+	2004-5, 2005-6, 2006-7
2C	–	+	–	+	–	+	+	+	2005-6
2D	+	+	–	+	+	+	–	+	2006-7, 2007-8, 2008-9
2E	+	+	–	–	–	+	+	+	2008-9, 2009-10, 2010-11, 2011-12, 2012-13
2F	–	+	–	–	–	+	+	+	2006-7, 2007-8, 2008-9
2G	–	+	–	–	–	+	–	+	2008-9
2H	–	+	–	–	–	–	–	+	2007-8
2I	+	+	–	–	+	+	–	+	2008-9
2J	+	+	–	–	–	+	–	+	2009-10, 2010-11
2K	+	+	–	–	–	–	+	+	2009-10
2L	+	–	–	–	–	+	+	+	2010-11
2 M	+	–	–	+	–	+	+	+	2010-11
2N	+	+	+	–	–	+	+	+	2010-11
2O	+	+	–	–	–	+	+	–	2011-12

a +, Position was detected as potentially *N*-glycosylated; –, position was not detected as *N*-glycosylated.

**TABLE 3:** Patterns of potential N-glycosylation sites for RSV-A sequence data from Kilifi, Kenya: genotype GA5

Designation code	Codon position^*a*^							Epidemic(s) observed
	103	105	238	242	250	273	294	
5A	+	+	+	–	+	+	+	2000-01, 2001-02, 2002-03-04, 2004-05, 2006-07
5B	+	+	+	–	–	+	+	2006-07
5C	+	+	+	+	+	+	+	2007-08

a +, Position was detected as potentially *N*-glycosylated; –, position was not detected as *N*-glycosylated.

Due to the G protein being characterized by a high prevalence of serine and threonine residues, and without the requirement of the Asn-X-Ser/Thr sequon, there were between 81 and 101 amino acid sites predicted to be *O*-glycosylated. As expected, the 24 additional amino acids in the genotype ON1 viruses offered more sites for potential *O*-glycosylation. The was also variation in the number and sites predicted to be *O*-glycosylated in successive epidemics as *N*-glycosylated, but the patterns were less clear and compounded by their ubiquity.

### Positive selection analysis.

Fifteen codon positions within genotypes GA2 and ON1 Kilifi sequences were observed to be under positive selection (ω > 1), selection that favors change in amino acids, with a posterior probability of >0.95; Table 4. These positively selected codon sites were 101, 104, 106, 115, 147, 225, 237, 244, 248, 250, 273, 274, 275, 286 (ON1: 310), and 289 (ON1: 313). This is despite the mean nonsynonymous/synonymous (*dN/dS*) substitution rate ratio (ω) for the analyzed region being <1, which indicates that generally the G glycoprotein is under purifying/negative selection (selection that disfavors change in amino acids). However, the M2a site model that accommodates positive selection among sites gave the highest likelihood score against the neutral and negative selection models (M0 and M1a, respectively). No sites were identified as positively selected among the GA5 sequences, probably due to the small numbers analyzed.

**TABLE 4:** Parameter estimates, *dN/dS*, log likelihood (ℓ), and positive selection sites for RSV-A sequence data from Kilifi, Kenya

Genotype and model	*dN/dS*^*a*^	Parameter estimates	Positively selected sites^*b*^	Log likelihood (ℓ)
GA2 and ON1				
M0	0.655	ω = 0.655	None	−3,681.07
M1a	0.603	p_0_ = 0.502, p_1_ = 0.498; ω_0_ = 0.21, ω_1_ = 1.00	Not allowed	−3,661.87
M2a	0.691	p_0_ = 0.496, p_1_ = 0.48, p_2_ = 0.03; ω_0_ = ,0.23 ω_1_ = 1.00, ω_2_ = 3.67	**274L**	−3,656.22
M3	0.712	p_0_ = 0.57, p_1_ = 0.41, p_2_ = 0.02; ω_0_ = 0.27, ω_1_ = 1.15, ω_2_ = 4.02	101F, 104L, 106G, 115L, 147T, **225V**, 237D, 244R, 248L, 250S, 273N, **274L**, 275S, **286L (ON1: 310)**, **289P (ON1: 313)**	−3,661.99
GA5				
M0	0.547	ω = 0.547	None	−1,261.92
M1a	0.547	p_0_ = 0.99, p_1_ = 0.01; ω_0_ = 0, ω_1_ = 1	Not allowed	−1,261.92
M2a	0.547	p_0_ = 1.00, p_1_ = 0.00, p_2_ = 0.00; ω_0_ = 0.55, ω_1_ = 1.00, ω_2_ = 1.00	None	−1,261.92
M3	0.547	p_0_ = 0.08, p_1_ = 0.92, p_2_ = 0.00; ω_0_ = 0.00, ω_1_ = 0.55, ω_2_ = 19.09	None	−1,261.92

a This *dN/dS* ratio is an average over all sites in the RSV-A alignment.

b The positively selected sites indicated in boldface are posterior probabilities >0.99. The positions are relative to the RSV-A reference strain (M74568).

### Relatedness of Kilifi and global RSV-A viruses.

**FIG 6:**
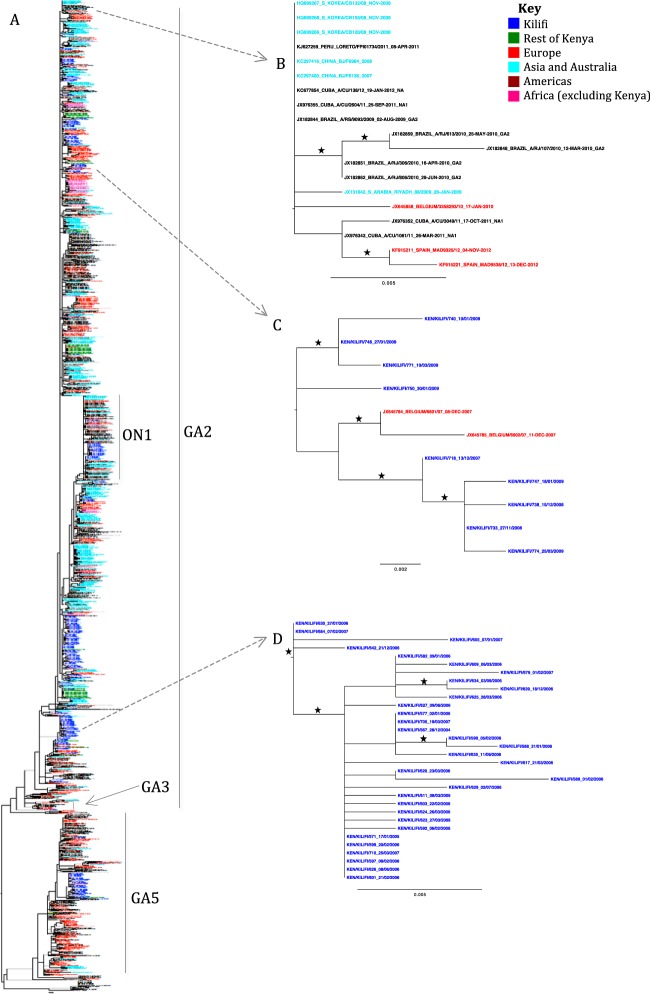
Phylogenetic placement of RSV-A viruses from Kilifi Kenya in the global context. (A) Global ML phylogenetic tree of 1,415 unique RSV-A G-gene sequences from 29 countries collected between 2000 and 2012. The taxon names for Kilifi sequences (*n* = 284) are in blue, while the rest of Kenya (*n* = 56) are in green. Sequences from other countries are color-coded as follows: red (Europe), cyan (Asia and Australia), black (Americas), and pink (Africa excluding Kenya). (B) Example of a cluster with variants detected abroad but not in Kilifi. (C) Example of a cluster with variants predominantly observed in Kilifi, with a few representatives from abroad. (D) Example of a cluster with variants only detected in Kilifi. Branches with bootstrap values of >60% are marked with a star (⋆).

The ML phylogenetic clustering of 1,415 global sequences from 29 countries is shown in Fig. 6A (enlarged version in Fig. S1 in the supplemental material). Kilifi viruses (blue colored taxa) were not placed into a single monophyletic group but in multiple clusters of various sizes along the tree. The composition of the clusters on the tree varied with most clusters having variants detected abroad but not in Kilifi (Fig. 6B), some clusters having variants predominantly observed in Kilifi with a few representatives from abroad (mainly Europe) (Fig. 6C), and some clusters with variants only detected in Kilifi (Fig. 6D).

All the clusters of global sequences fell within the genotypes detected in Kilifi except one cluster comprising of sequences from the United States only from 2003 to 2007 that seemed to be off the main GA5 branch. Most of the global viruses, as was the case in Kilifi, were of genotype GA2.

## DISCUSSION

The mechanisms underlying recurrent RSV epidemics have long been studied but are still poorly understood. Comparative analysis of time-stamped molecular sequence data is essential for reconstructing the genetic history of RSV and inferring the nature and extent of forces shaping the evolution of the virus that may play a role in the recurrent epidemics. This report details the genetic diversity and molecular evolution of RSV group A viruses through a long-term surveillance of RSV at the Kenyan coast, a tropical, largely rural developing country setting.

As reported elsewhere (47, 48), we observed a change from multiple group A genotype circulation in Kilifi to predominance of genotype GA2 over the 12 years studied. Nonetheless, there was continued diversification within the dominant genotype GA2 evidenced via (i) increased number of distinct variants over time within this genotype and (ii) proposed designation of some groups within GA2 into new genotypes, such as NA1 and NA2 (49). There appears to be a replacement period of around 7 years for some genotypes to appear, predominate, and then become undetectable—in this case GA5, then GA2, and then ON1. It could be the period it takes to build up sufficient herd immunity to a genotype through infection across all ages and thus enable replacement (at the population level) by a less-well-recognized genotype.

Phylogenetic analysis revealed ML trees with clusters of viruses derived from single and multiple epidemics, i.e., some variants were observed within a single epidemic while others were detected across several epidemics (2 to 5 years). However, persistent variants were very few comprising only 22.5% of the total number of variants assigned. It was also clear from the ML trees that individual epidemics were driven by multiple variants characterized by strains from the same epidemic placed in multiple clusters. Only two variants were seen to reappear after nondetection in consecutive epidemic seasons. Similar observations of multiple variants seeding epidemics, with little persistence between consecutive epidemics and limited variant reemergence have been reported for group B RSV from Kilifi (9). Although it remains to be understood whether multiple variants is an absolute necessity for or an occurrence of community epidemics, it is also characteristic for other seasonal respiratory viruses such as influenza (50, 51).

Similarly, the global phylogeny showed that most countries had multiple cocirculating sequence clusters from a single year. The RSV-A viruses from Kilifi often clustered closest to viruses from other regions around Kenya, which points to within-country transmission playing a pivotal role in local RSV persistence. At the global scale, however, Kilifi viruses clustered closest with viruses from Europe. Since Kilifi lies on the Kenyan coast and is a popular tourism destination especially for visitors from Europe, these findings are consistent with an argument that Europe could be a direct source of new RSV variants into Kilifi and for Kenya in general. However, more extensive sampling regionally around Kenya will point to the most probable source(s) of variants into Kilifi as there are limited sequences from Africa (only sequences from Kenya and South Africa were available in this analysis). Lastly, with many clusters on the tree not having representatives from Kilifi or Kenya, it is evident that there are many RSV-A variants circulating globally but not observed locally.

While the evolutionary rate of a virus provides a clue on its adaptive potential in a new host, the demographic history highlights changes in its relative genetic diversity over time (52). The changes in relative dynamic diversity were characterized by expansions and contractions coinciding with RSV cases peaks and troughs, respectively. Further, the major peaks in genetic diversity coincided with the epidemics that had the highest number of RSV A variants. The fact that the demographic history could capture changes in population size at fine temporal resolution implies sufficient sampling density. The mean substitution rate for the Kilifi viruses together with contemporaneous global data set was estimated at 3.06 × 10^−3^ nucleotide substitutions/site/year, which was slightly higher than previous global estimates (1.83 × 10^−3^ [35]), and these differences in estimates could result from differences in the source of isolates, the length of the G sequence used and the estimation method (53). The TMRCA analysis gives an upper bound to the date of the emergence of a pathogen assuming a single initial case, and from this analysis we have estimated that the MRCA of the global RSV A strains collected between 2000 and 2012 from 29 countries existed at or before 1968, similar to a previous estimate for RSV A whole genomes collected between 2001 and 2011 (54).

In this analysis, we identified 34 codon sites with amino acid substitutions that were distinct between genotypes for the group A viruses identified within Kilifi. However, a large proportion of these sites had an amino acid shared between genotypes with only three sites (250, 297, and 298) having a unique amino acid for each genotype. The sharing pattern indicates the genetic relatedness between these genotypes; GA2 and ON1 were most closely genetically related to GA3, whereas GA5 and GA3 were the most distantly related. It is interesting that previous studies have described amino acid changes at the other detected genotype defining sites 226 and 274 for RSV isolates that have lost strain-specific and group-specific epitopes (6, 55, 56).

Specific positions in the G protein have been shown to be under adaptive evolution (35, 57). We identified 15 positively selected codon sites within the two hypervariable mucin-like regions of the G protein. All of these sites have similarly been reported as positively selected, as summarized by Trento et al. (58). Amino acid replacements in sites 225, 274, and 275 have been previously described in escape mutants, selected with specific monoclonal antibodies and in natural isolates (59–61). In addition, sites 225, 274, and 286 have been described to have codon/amino acid reversal tendencies, otherwise known as the “flip-flop” pattern. Notably, only 6.5% (15/230) of the codon sites analyzed were detected as adaptive, in agreement with an average *dN*/*dS*<1, despite 44.6% (308/690) of the nucleotide sites being variable. This could imply that most of the substitutions are either neutral or responsible for sustained transmission of the virus through other mechanisms.

*N*-glycosylation is important for viral cell-cell and cell-extracellular matrix attachment (62, 63), protein folding (64), immunological properties (65–67), and stability (68). We detected eight and seven potential *N*-glycosylation sites within genotypes GA2/ON1 and GA5 sequences, respectively, with varied patterns between different epidemics. Sites 103, 135, 251, and 294 have been similarly detected as *N*-glycosylated in previous studies (53). Interestingly, no single pattern was observed continuously for the 12 years of the study period for the GA2 and ON1 sequences, with the majority of the patterns only seen in single epidemics. Similarly, none of GA5 *N*-glycosylation patterns was observed for the entire period of GA5 detection in Kilifi. There were >8-fold more potential *O*-glycosylated sites as *N*-glycosylated sites, but with similar variation in the number and sites over successive epidemics. The variations in patterns of *O*- and *N*-glycosylation were concurrent with progressive amino acid variation within the sequences analyzed. It is thought that such a high degree of variability in amino acid composition is important for molecular adaptation and is characteristic of relaxed selective constraints within these regions (69). Changes in *N*-glycosylation sites in influenza have been reported to result in marked decrease in reactivity with human sera (70) or increased virulence in seasonal influenza (71). Previous studies in RSV have indicated that epitopes within the C terminus of G-protein fragments can be masked by glycans from reactivity to certain sera, with corresponding reactivity to their nonglycosylated forms (22). Three of the 12 *N*-glycosylated sites were detected as positively selected. It is therefore plausible that these changing patterns of *N*-glycosylation sites are suggestive of a mechanism by which the virus evades preexisting immune responses.

There are conflicting views as to the impact of the variability of the ectodomain of the G protein on its reaction with antibodies and as to whether antibody selection is involved in virus evolution. Trento et al. (58) reported a high level of conservation of G protein epitopes over time, as determined by the reactions of “strain-specific” monoclonal antibodies with recent RSV isolates, and concluded that antibody-driven selection was not significant. However, an alternative explanation to their observations is that the “strain-specific” antibodies were mostly specific to genotypes rather than strains or variants. It has previously been shown that single amino acid changes can abrogate the reaction of human convalescent-phase sera with peptides whose sequences are based on naturally occurring amino acid sequences (59). For example, the change of codon 287 from proline to leucine could completely abrogate a baby's convalescent-phase serum reaction with a peptide encompassing codons 283 to 291. In addition, all the peptides found to react with babies' convalescent-phase sera comprised regions of the G protein that had potential *N*-glycosylation sites in at least some isolates. In view of the considerable variability of potential *N*-glycosylation observed within GA2, it may be that single nucleotide changes could have profound effects on the antigenicity of the G protein.

In conclusion, our analysis shows that RSV is a continually evolving virus whose persistence in the community is driven by evolutionary factors (nucleotide and amino acid variability, variation in *N*-glycosylation site patterns and selection) and epidemiological factors (multiple strain introductions and recurrence). Similar analysis with whole-genome data will illuminate further on the genetic and epidemiological characteristics of the virus and vaccine development strategies.

## Supplementary Material

Supplemental material
