# Supplementary material for: Molecular Evolutionary Dynamics of Respiratory Syncytial Virus Group A in Recurrent Epidemics in Coastal Kenya
Source: J Virol. 2016 Apr 29;90(10):4990–5002. doi: 10.1128/JVI.03105-15 (PMC4859726; doi:10.1128/JVI.03105-15)
Supplement: Supplemental material [file supp_90_10_4990__index.html]

Molecular Evolutionary Dynamics of Respiratory Syncytial Virus Group A in Recurrent Epidemics in Coastal Kenya — Supplemental material 

# Molecular Evolutionary Dynamics of Respiratory Syncytial Virus Group A in Recurrent Epidemics in Coastal Kenya

## Supplemental material

- Supplemental file 1 -

  Table S1 (Frequency of IFAT- and PCR-diagnosed RSV cases and number sequenced over 13 successive epidemics at Kilifi, Kenya.)

  Fig. S1 (Phylogenetic placement of RSV-A viruses from Kilifi, Kenya, in the global context.)

  PDF, 325K
