# Supplementary material for: Molecular Evolutionary Dynamics of Respiratory Syncytial Virus Group A in Recurrent Epidemics in Coastal Kenya
Source: J Virol. 2016 Apr 29;90(10):4990–5002. doi: 10.1128/JVI.03105-15 (PMC4859726; doi:10.1128/JVI.03105-15)
Supplement: Supplemental material [file JVI.03105-15_zjv999091626so1.pdf]

Molecular evolutionary dynamics of respiratory syncytial virus group A in recurrent epidemics in coastal Kenya

James R. Otieno[1#], Charles N. Agoti[1, 2], Caroline W. Gitahi[1], Ann Bett[1], Mwanajuma Ngama[1], Graham F Medley[3], Patricia A. Cane[4] and D. James Nokes[1, 5]

Authors' affiliations

1. *Department of Epidemiology and Demography, Kenya Medical Research Institute (KEMRI) – Wellcome Trust Research Programme, Kilifi, Kenya*
2. *Department of Biomedical Sciences, Pwani University, Kilifi, Kenya*
3. *Department of Global Health & Development, London School of Hygiene and Tropical Medicine, London, UK*
4. *Public Health England, Salisbury, UK*
5. *School of Life Sciences and WIDER, University of Warwick, Coventry, UK*

#CORRESPONDENCE

jotieno@kemri-wellcome.org

Table S1: Frequency of IFAT and PCR diagnosed RSV cases and number sequenced over 13 successive epidemics at Kilifi, Kenya.

**Epidemic[ϕ] Season**	**Number of RSV Positive samples**	**Number (%) of RSV A samples**	**Number (%) of RSV-A samples Sequenced[*]**	**Number (%) of RSV-B samples**	**Number (%) of RSV A+B samples**	**Number (%) of unique Sequences[β]**
2012 - 2013	152	110 (72.4)	53 (48.1)	27 (17.8)	3 (2)	22 (41.5)
2011 - 2012	161	55 (34.2)	52 (94.5)	102 (63.4)	2 (1.2)	26 (50.0)
2010 - 2011	279	241 (86.4)	158 (65.6)	21 (7.5)	7 (2.5)	72 (45.6)
2009 - 2010	259	120 (46.3)	88 (73.3)	95 (36.7)	9 (3.5)	33 (37.5)
2008 - 2009	208	154 (74)	47 (30.5)	41 (19.7)	0 (0)	29 (61.7)
2007 - 2008	256	33 (12.9)	20 (60.6)	197 (77)	3 (1.2)	14 (70.0)
2006 - 2007	195	153 (78.5)	81 (52.9)	22 (11.3)	1 (.5)	32 (39.5)
2005 - 2006	239	224 (93.7)	56 (25.0)	9 (3.8)	0 (0)	21 (37.5)
2004 - 2005	183	46 (25.1)	11 (23.9)	119 (65)	3 (1.6)	5 (45.5)
2003 - 2004	114	68 (59.6)	7 (10.3)	16 (14)	0 (0)	6 (85.7)
2002 - 2003	89	42 (47.2)	25[#]	35 (39.3)	0 (0)	8 (32.0)

2000 - 2001	N/A	N/A	19[σ]	N/A	N/A	10 (52.6)
**Total**	**2135**	**1246 (58.4)**	**658 (46)**[γ]	**684 (32.0)**	**28 (1.3)**	**293 (44.5)**

[ϕ] An epidemic season defined by the time period between September of one year to August of the following year.

Highlighted in grey are the RSV-A dominated epidemics. However, we do not know for 2000-2001 and 2001-2002 which group was dominant as full surveillance was not established.

[*] Number sequenced as a percentage of those typed to be RSV-A.

[β] Unique sequences defined as sequences that differ by at least one nucleotide.

[σ] Percentage indeterminable as we don't have data on number of diagnosed RSV cases at the hospital during this time.

[#] Percentage indeterminable as we only have sequences from a birth cohort in this epidemic and not the inpatient surveillance sequences.

[γ] The total number of RSV-A sequenced includes 633 sequences from inpatient and outpatient RSV surveillance period (2000-2012), and 25 Kilifi Birth Cohort sequences for the 2002/2003 epidemic, i.e. 633 + 25 = 658, while the percentage is based only on the inpatient surveillance 573/1246.

Fig. S1: Phylogenetic placement of RSV-A viruses from Kilifi, Kenya, in the global context. This is a global Maximum Likelihood phylogenetic tree of 1,415 unique RSV-A sequences from 29 countries collected between 2000 and 2012. The taxon names for Kilifi sequences (*n=284*) are in blue while the rest of Kenya (*n=56*) in green. Sequences from other countries are colour-coded as follows: Red (Europe), Cyan (Asia and Australia), Black (Americas) and Pink (Africa excluding Kenya). Branches with bootstrap values >60% are shown with a star (★).

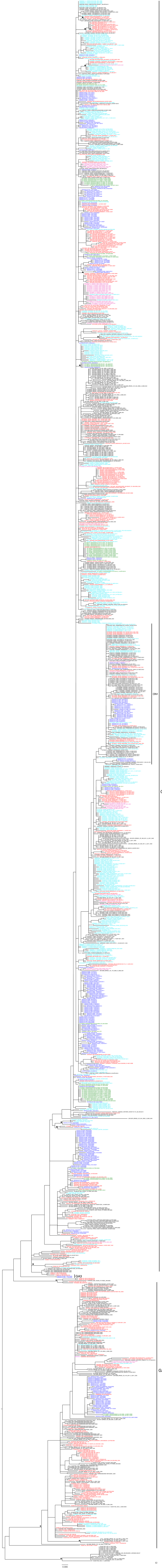

ON1
GA2
GA3
GA5
0.005
